# Pentosan polysulfate induces low-level persistent prion infection keeping measurable seeding activity without PrP-res detection in Fukuoka-1 infected cell cultures

**DOI:** 10.1038/s41598-022-12049-z

**Published:** 2022-05-13

**Authors:** Hanae Takatsuki, Morikazu Imamura, Tsuyoshi Mori, Ryuichiro Atarashi

**Affiliations:** Division of Microbiology, Department of Infectious Diseases, Faculty of Medicine, University of Miyazaki, 5200 Kihara, Kiyotake-cho, Miyazaki, 889-1692 Japan

**Keywords:** Biochemistry, Drug discovery, Diseases of the nervous system

## Abstract

Each prion strain has its own characteristics and the efficacy of anti-prion drugs varies. Screening of prion disease therapeutics is typically evaluated by measuring amounts of protease-resistant prion protein (PrP-res). However, it remains unclear whether such measurements correlate with seeding activity, which is evaluated by real-time quaking-induced conversion (RT-QuIC). In this study, the effects of anti-prion compounds pentosan polysulfate (PPS), Congo red, and alprenolol were measured in N2a58 cells infected with Fukuoka-1 (FK1) or 22L strain. The compounds abolished PrP-res and seeding activity, except for N2a58/FK1 treated with PPS. Interestingly, the seeding activity of N2a58/FK1, which was reduced in the presence of PPS, was not lost and remained at low levels. However, upon removal of PPS, both were gradually restored to their original levels. These results indicate that low-level persistent prion infection keeping measurable seeding activity is induced by PPS in a strain-dependent manner. Furthermore, for protein misfolding cyclic amplification (PMCA), the anti-prion effect of PPS decreased in FK1 compared to 22L, suggesting that the differences occur at the level of the direct conversion. Our findings demonstrate that the advantages of RT-QuIC and PMCA can be exploited for more accurate assessment of therapeutic drug screening, reflecting strain differences.

## Introduction

Prion disease is a fatal neurodegenerative disease caused by the accumulation of abnormal prion protein (PrP^Sc^), a structurally altered form of normal prion protein (PrP^C^), in the central nervous system. Human prion diseases include Creutzfeldt–Jakob disease (CJD), Gerstmann–Strässler–Schenker syndrome (GSS), and fatal familial insomnia, while animal prion diseases include bovine spongiform encephalopathy (BSE) in cattle, scrapie in sheep, and chronic wasting disease in deer. Some regions of PrP^Sc^ are protease-resistant and remain after proteinase K (PK) treatment, which can be detected by Western blotting (WB) as protease-resistant PrP^Sc^ (PrP-res)^1^.

The main therapeutic targets of prion diseases are inhibition of PrP-res production, promotion of its degradation, and reduction of its neurotoxicity for early-stage cases, as well as regeneration of neural tissue for advanced cases. To screen for therapeutic agents for prion diseases targeting inhibition of PrP-res production and promotion of its degradation, candidate compounds are often added to cultured cells infected with scrapie strains, and the effect on PrP-res levels is examined by WB. Various anti-prion compounds have been found to reduce PrP-res levels in cultured cells and infected animals^2,3^. For example, pentosan polysulfate (PPS)^4^, Congo red^5^, and alprenolol^6^ can reportedly reduce PrP-res levels in prion-infected cultured cells. Among such drugs, PPS reduced accumulation of PrP^Sc^ in the brain and/or prolonged survival in prion-infected mice^7–9^. In addition, PPS has been used in clinical practice for variant CJD and other prion diseases, although the number of cases was limited^10,11^. To date, no effective prophylactic or therapeutic agent for prion diseases has been identified in clinical trials.

Detection of PrP-res by WB may be insufficient for use of PrP-res as an index of anti-prion effects (in terms of detection sensitivity) because prions can remain even after PrP-res disappears. Therefore, we developed a highly sensitive in vitro prion-detection method, the real-time quaking-induced conversion (RT-QuIC) assay^12^. RT-QuIC uses *Escherichia coli*-derived recombinant PrP as a substrate to promote the conversion reaction by agitation in samples suspected of containing prions (seed), and the process is measured as the fluorescence intensity of thioflavin T to determine the presence of prions^13^. Numerous reports show the usefulness of RT-QuIC for diagnosis of prion diseases in humans and animals using cerebrospinal fluid and other specimens^14^. In combination with a serial dilution method, RT-QuIC can also measure the seeding activity as a 50% seeding dose (SD_50_)^15^ with more than 10^3^-fold higher sensitivity than detection of PrP-res by WB^16^. Therefore, RT-QuIC may be more useful than WB for measuring anti-prion effects^17^. The purpose of this study was to measure the effects of anti-prion compounds on seeding activity using the RT-QuIC method and compare the results with PrP-res levels to determine whether RT-QuIC can improve screening for prion disease drugs.

## Results

### Seeding activity of cell cultures persistently infected with prions can be measured by RT-QuIC

**Figure 1:**
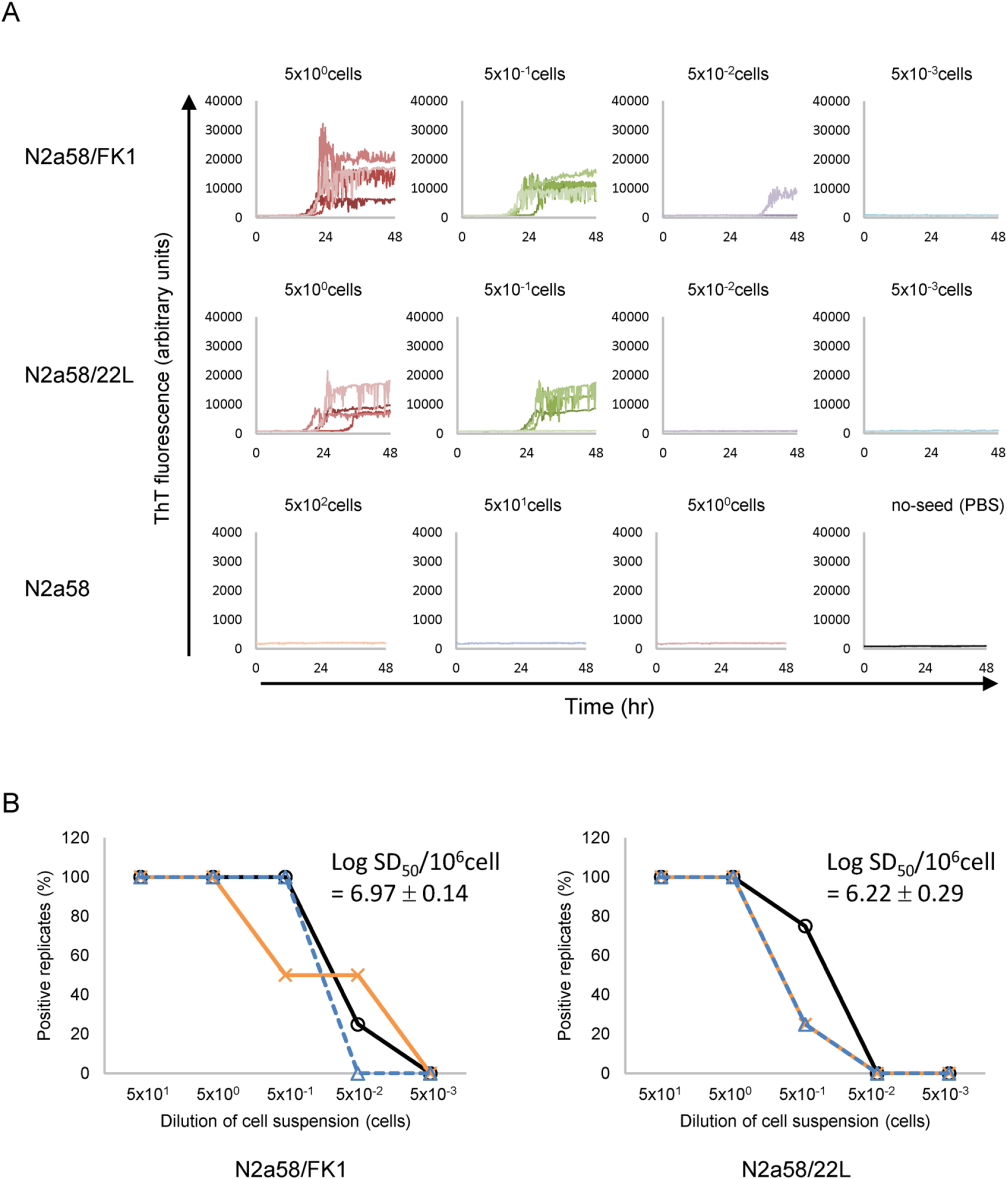
Quantification of seed activity in prion-infected cells using Endpoint RT-QuIC. (**A**) RT-QuIC assays were performed with serial dilutions of N2a58/FK1, N2a58/22L, and N2a58 cell lysates (4 wells/each dilution). Representative results are shown. (**B**) Positive response rates for RT-QuIC at each dilution in three independent experiments are shown in the graphs; Log SD_50_/10^6^ cells (mean ± standard deviation) was calculated by the Spearman-Kärber method. Positive results were considered when the fluorescence value of ThT increased more than twofold above the reference value.

To first determine the seeding activity of prion-infected cells, suspensions of N2a58 cells persistently infected with the FK1 or 22L strain were used for quantification by the Endpoint RT-QuIC method (Fig. 1). RT-QuIC reactions were performed in four independent wells using each diluted cell suspension as a seed. No increase in thioflavin T fluorescence was observed in uninfected N2a58 cells or without cell seeding (PBS only), whereas an increase in fluorescence was observed relatively quickly in all wells when more than 5 prion-infected cells were added (Fig. 1A). To clearly quantify seeding activity, the RT-QuIC reaction was performed three times and the mean value was calculated to determine the 50% seeding dose per 10^6^ cells (SD_50_/10^6^ cells) (Fig. 1B). Log SD_50_/10^6^ cells values for N2a58/FK1 and N2a58/22L were 6.97 ± 0.14 and 6.22 ± 0.29, respectively (Table 1).

**Table 1:** Seeding activity in anti-prion compounds treated prion-infected cells.

Passage No.	Log SD50/10^6^cell (mean ± standard deviation)							
	Untreated		Congo red		Alprenolol		PPS	
	N2a58/FK1	N2a58/22L	N2a58/FK1	N2a58/22L	N2a58/FK1	N2a58/22L	N2a58/FK1	N2a58/22L
0	6.97 ± 0.14	6.22 ± 0.29	–	–	–	–	–	–
1	–	–	6.05 ± 0.25	5.47 ± 0.72	6.30 ± 0.43	5.38 ± 0.29	6.22 ± 0.14	4.63 ± 0.14
2	–	–	4.80 ± 0.43	4.47 ± 0.38	4.63 ± 0.52	< 3.80	5.47 ± 0.14	< 3.38
3	–	–	< 3.22	< 3.3	< 3.80	< 3.55	4.47 ± 0.14	–
6	–	–	–	–	–	–	5.13 ± 0.52	–
9	–	–	–	–	–	–	4.72 ± 0.29	–

### N2a58/FK1 treated with PPS exhibited persistent seeding activity

**Figure 2:**
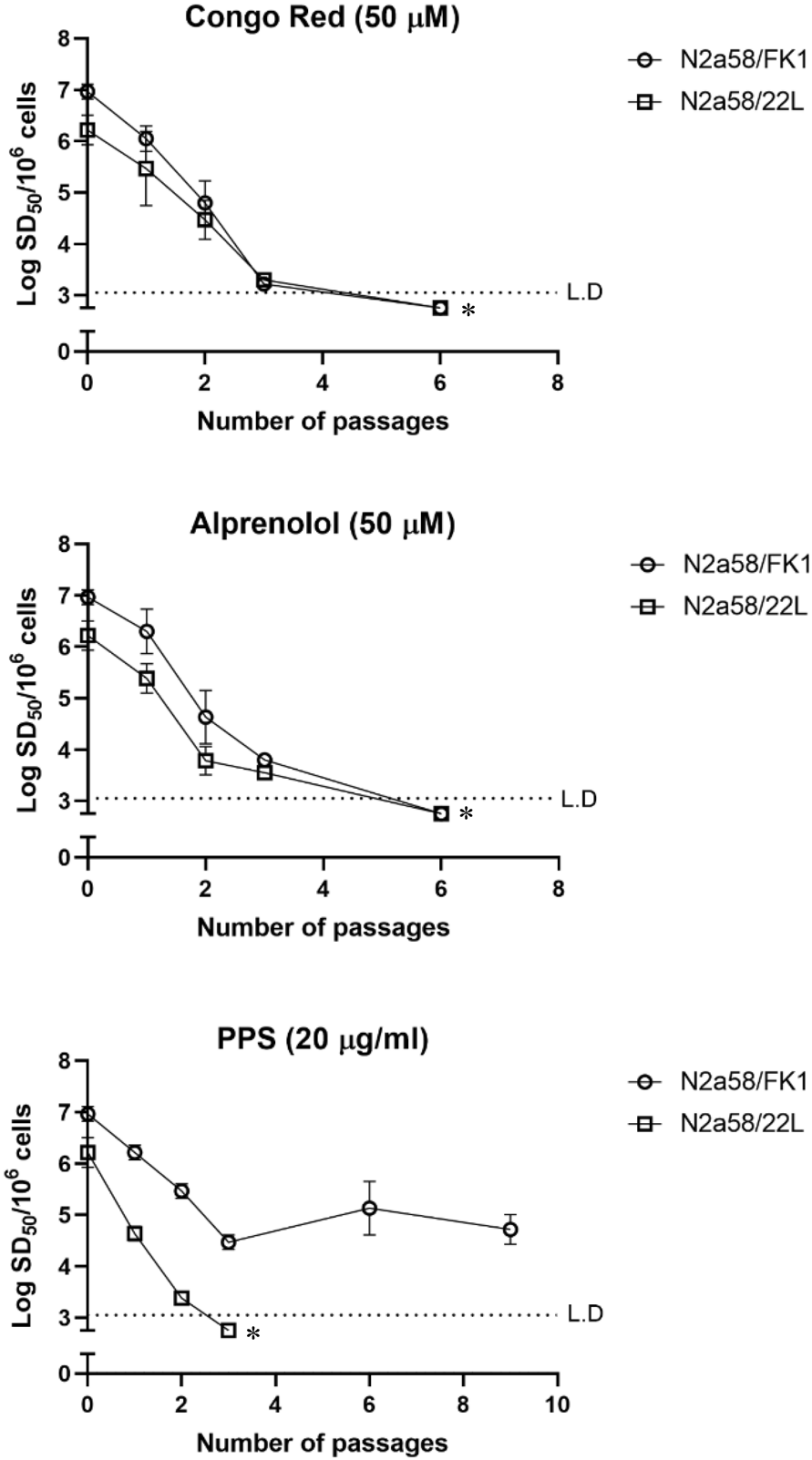
Seeding activity in prion-infected cells treated with anti-prion compounds. Prion-infected N2a58 cells were treated with Congo Red, Alprenolol, or PPS from P0 to P6. For each passage, prion seeding activity (Log SD_50_/10^6^ cells) was quantified from three independent experiments using RT-QuIC (mean ± standard deviation). Open circles and squares indicate N2a58/FK1 and N2a58/22L, respectively. Asterisks indicate less than the limit of detection (L.D.).

Seeding activities of N2a58/FK1 and N2a58/22L cultured in the presence of Congo red, alprenolol, or PPS (which have reported anti-prion effects) were quantified by RT-QuIC. Seeding activities of prion-infected cells treated with Congo red or alprenolol both decreased and became below the detection limit after the sixth passage (P6) (Fig. 2 and Table 1). In the presence of PPS, the seeding activity of N2a58/22L was below the detection limit after P3, but interestingly, that of N2a58/FK1 remained at 4.72 ± 0.29 log SD_50_/10^6^ cells even after P9 (Fig. 2 and Table 1). Amounts of PrP-res in PPS-treated prion infected cells were decreased in both 22L and FK1 strains, and almost disappeared at P2 and P3 (Fig. 4A). In contrast, no obvious change was observed for total PrP after PPS treatment.

**Figure 3:**
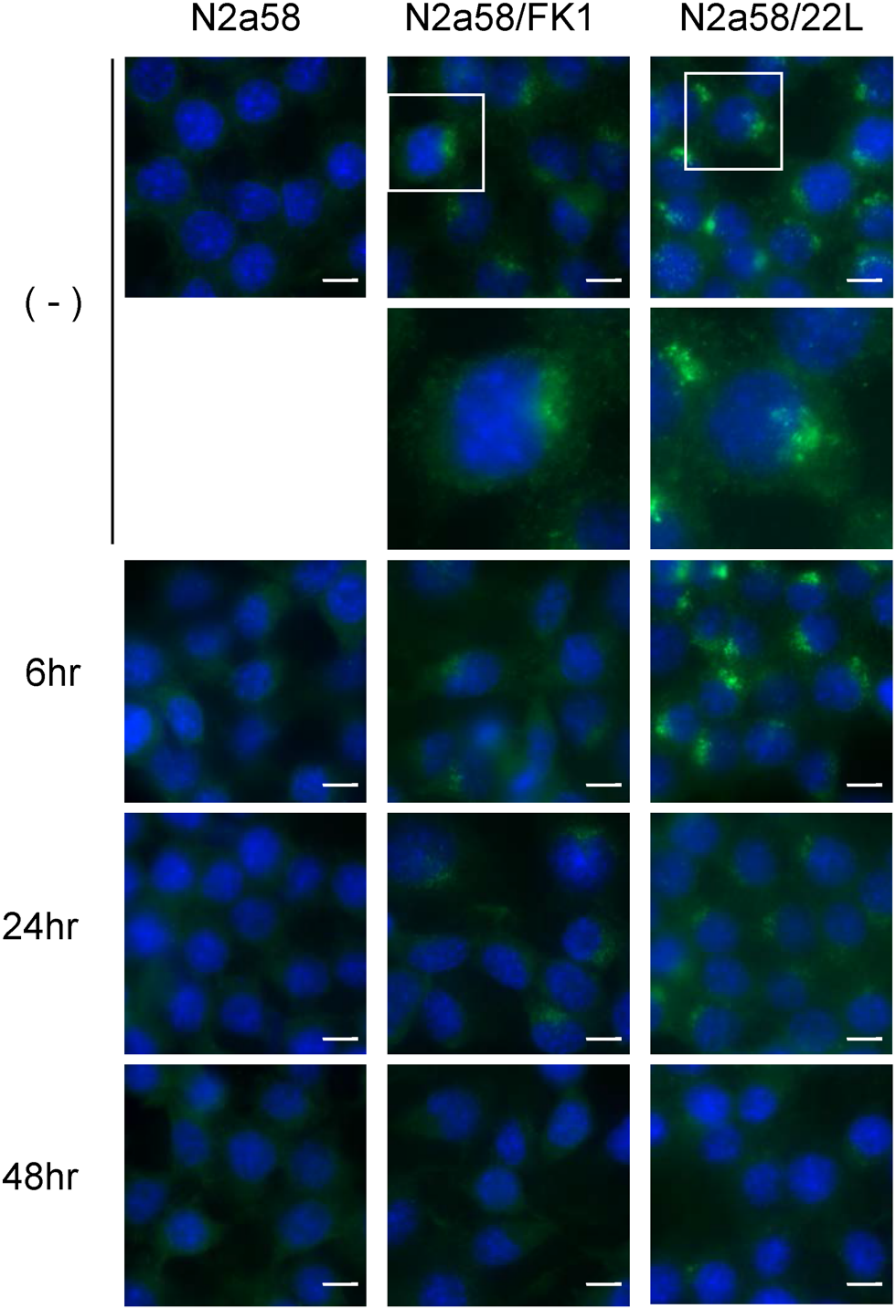
Influence of PPS on intracellular PrP^Sc^. Prion-infected N2a58 cells were cultured with 20 μg/ml of PPS for 6, 24, or 48 h. (−) indicates no PPS treatment. Panels show merge image of PrP^Sc^ (green) and nuclei (blue). Scale bar: 10 μm.

The time course (after 6, 24, and 48 h) of PrP^Sc^ signals of N2a58, N2a58/FK1, and N2a58/22L in the presence of PPS was observed by immunofluorescence. The signal intensity of N2a58/FK1 began to decrease after 6 h of PPS treatment, followed by a clear decrease in both N2a58/FK1 and N2a58/22L cells after 24 h (Fig. 3). The signals were indistinguishable from that of uninfected cells after 48 h (Fig. 3).

### Seeding activity and PrP-res of N2a58/FK1 were restored to original levels following PPS removal

**Figure 4:**
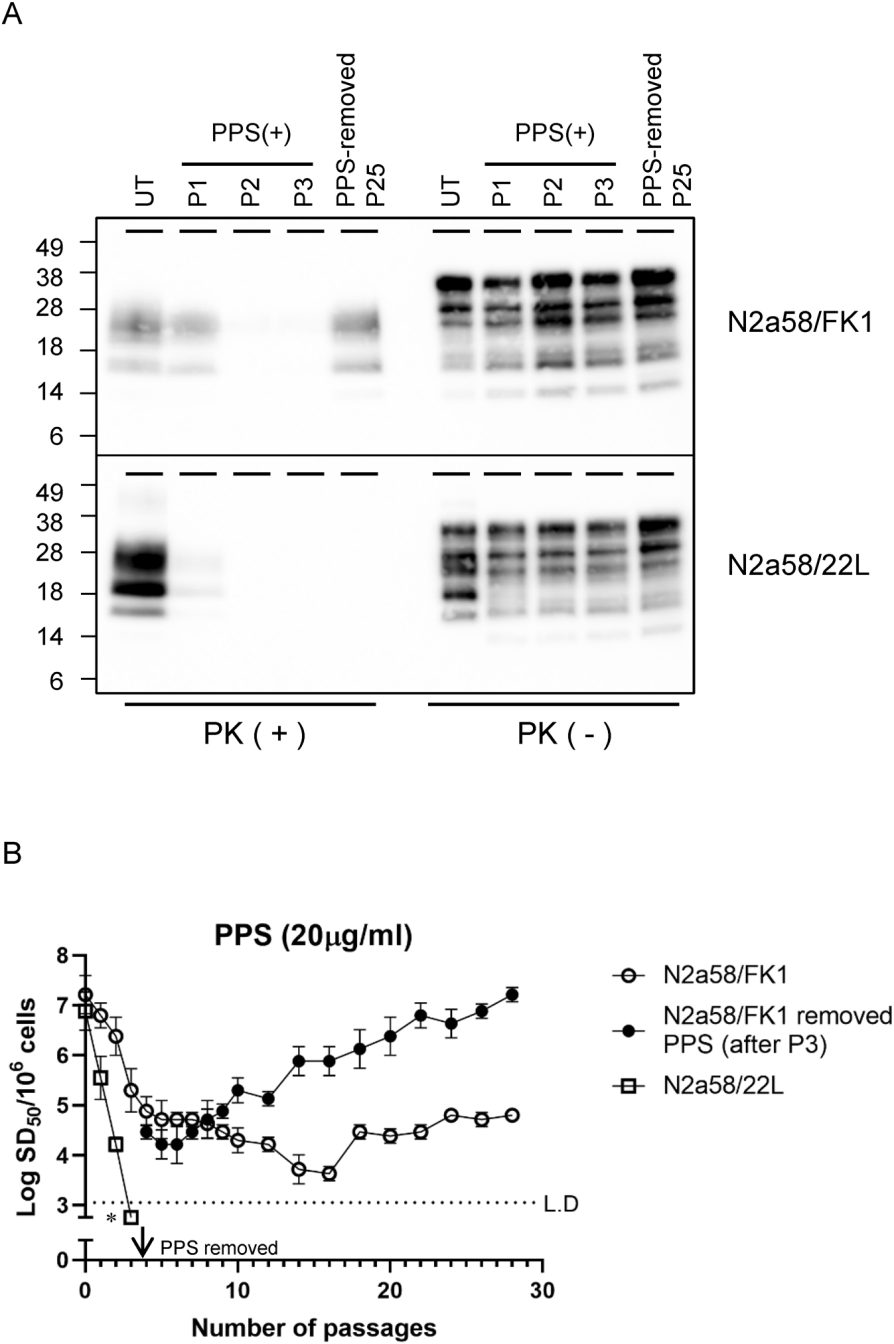
Timecourse of PrP-res levels and seeding activity of prion-infected cells treated with PPS continuously or removed after P3. (**A)** PrP-res [PK( +)] and total PrP [PK(−)] were analyzed by WB in P1–P3 prion-infected cells cultured in the presence of PPS and P25 prion-infected cells after excluding PPS. Total protein content of N2a58/FK1 cell lysate per lane was 60 μg and 20 μg for PK( +) and PK(−), respectively; for N2a58/22L, 45 μg and 15 μg were used for PK( +) and PK(−), respectively. UT represents untreated cells. The full-length blot image is provided in the Supplementary Fig. 1. **(B)** For each passage, prion seeding activity (Log SD_50_/10^6^ cells) was quantified from three independent experiments using RT-QuIC (mean ± standard deviation). Open circles and open squares indicate N2a58/FK1 with PPS and N2a58/22L with PPS, respectively. Closed circles indicate N2a58/FK1 cultured in medium without PPS after P3. Asterisk indicates below limit of detection (L.D.).

PPS efficiently abolished PrP-res in both N2a58/22L and N2a58/FK1. However, low levels of seeding activity (3–5 log SD_50_/10^6^ cells) were maintained in N2a58/FK1 even after P28 in the presence of PPS (Fig. 4B). Therefore, we investigated whether the decrease in seeding activity elicited by PPS could be recovered by removal of PPS from the culture medium. After P4, prion-infected cells were cultured in medium without PPS. Thereafter, the seeding activity of N2a58/FK1 gradually increased such that by P25, seeding activity and PrP-res levels had returned to initial levels (Fig. 4A,B). In contrast, seeding activity and PrP-res did not recover in N2a58/22L (Fig. 4A).

### Inhibitory effect of PPS on the PrP^Sc^ conversion reaction in PMCA varied greatly among prion strains

**Figure 5:**
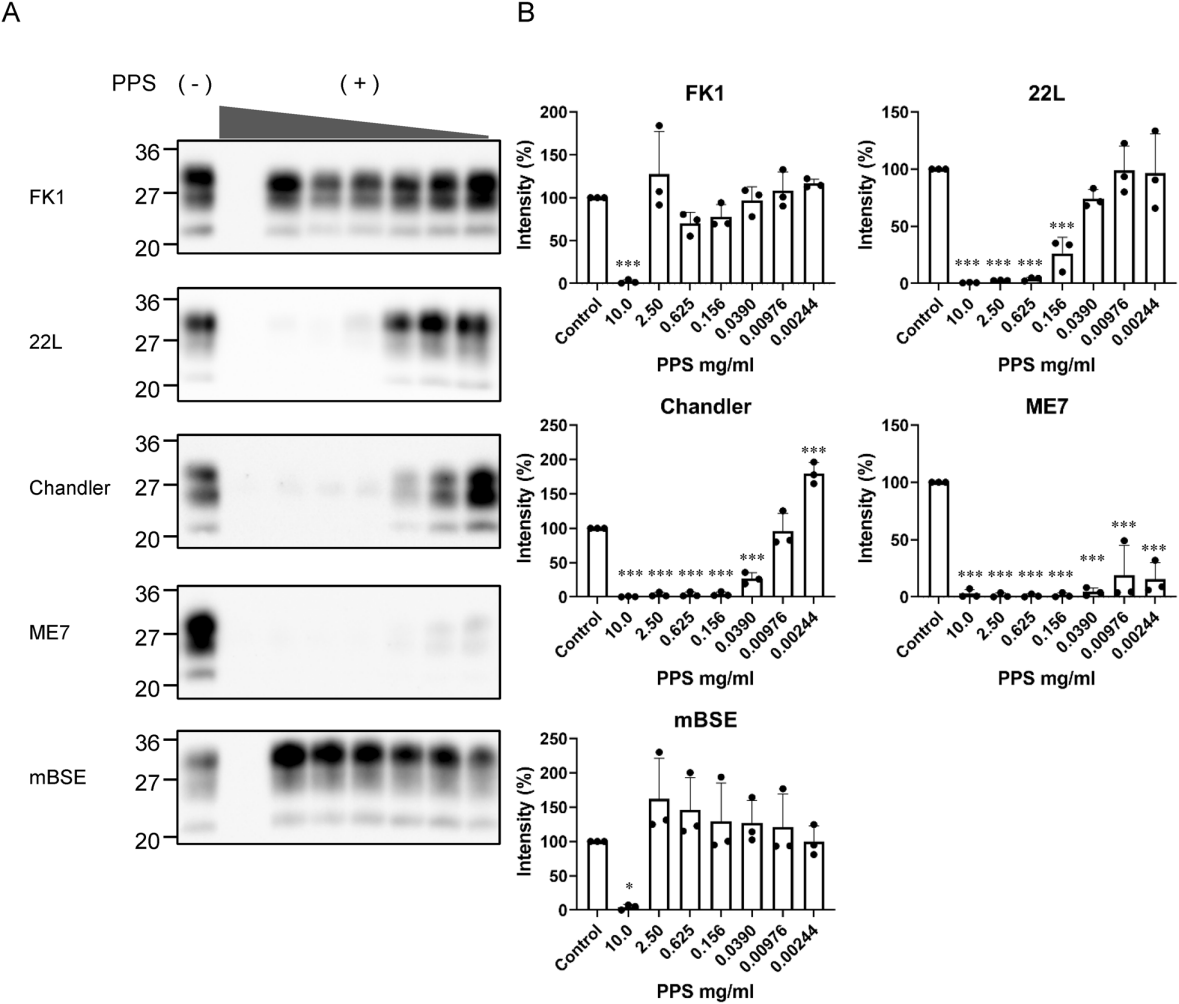
Inhibitory effect of PPS on PMCA. **(A)** Brain homogenates of prion-infected mice were used as seeds for PMCA with a four-fold serial dilution of PPS (10 mg/ml, 2.5 mg/ml, 0.625 mg/ml, 0.156 mg/ml, 39 μg/ml, 9.8 μg/ml, and 2.4 μg/ml). Representative images of WB of PrP-res are shown. The corresponding full-length blot images, and blots for each of the three independent PMCA experiments are provided in the Supplementary Figs. 2–5. (**B)** For each PPS concentration, the intensity of PrP-res was quantified from three independent experiments (mean ± standard deviation). Control represents PMCA without PPS and is set at 100%. Statistical analysis was performed using one-way ANOVA followed by Dunnett’s test. *p < 0.05, **p < 0.01, ***p < 0.001 compared with control.

We next used the PMCA method to examine whether PPS directly inhibited the PrP^Sc^ conversion reaction and whether this inhibitory effect differed among prion strains. PMCA is a method to promote the conversion reaction by fragmenting^18,19^ or depolymerizing PrP multimers^20^ by intermittent sonication using PrP^C^ in brain homogenate as a substrate^18^. Compared with RT-QuIC, PMCA can amplify PrP^Sc^ without being significantly affected by solvents such as dimethyl sulfoxide. In fact, the anti-prion effect of cationic tetrapyrrole dissolved in dimethyl sulfoxide using PMCA has been reported^21^. PPS was added to the reaction mixture at a four-fold dilution from a final concentration of 10 mg/ml to 2.44 μg/ml, and five prion strains (FK1, 22L, Chandler, ME7, and mBSE) were used as seeds. Inhibition of the PrP^Sc^ conversion reaction of PPS was strongest in ME7 (significant decrease at 2.44 μg/ml or higher), followed by Chandler (39 μg/ml or higher) and 22L (156 μg/ml or higher), but occurred in FK1 and mBSE only at 10 mg/ml (Fig. 5).

## Discussion

In the present study, we found that even after PPS elicited the disappearance of PrP-res from N2a58/FK1, RT-QuIC results indicated that a measurable level of seeding activity remained despite passaging 28 times in the presence of PPS. Furthermore, this residual seeding activity and loss of PrP-res recovered to original levels after PPS removal, indicating that the FK1 strain continues to grow at levels below the PrP-res detection limit in the presence of PPS. The concentration of PPS administered to cultured cells in this study (20 μg/ml) was higher than those used in some previous reports using prion-infected cultured cells^22–24^. In addition, the efficacy of antiprion drugs would be expected to increase inversely correlated with a decrease in the amount of PrP-res itself in the cells. Thus, it can be assumed that PPS is present in excess relative to the amount of PrP-res, which has decreased below detectable levels after PPS administration in the FK1-infected cells. Although we cannot completely rule out the possibility that the PPS concentration did not reach a level that could completely suppress the seeding activity of the FK1 strain, the fact that seeding activity was not lost even in the presence of excessive amounts of PPS indicates that it is unlikely that the concentration of PPS used was not sufficient.

Previously, it was known that latent prion infections between heterologous animals occur in what is often referred to as a "carrier state," in which PrP-res accumulation and seeding activity is present in the host body but does not cause disease^25^. For example, in a study conducted in the United Kingdom (UK) investigating the presence of variant CJD carriers in 32,441 appendix samples, 16 were positive for PrP-res^26^. It would be interesting to see how many of these samples would be positive when examined by RT-QuIC. It has also been reported that seeding activity can remain for long periods of time even in PrP knockout mice^27^. Although it should be cautious to compare the phenomenon in cultured cells in this study to latent prion infection at the individual level, it is important to better understand the pathological significance of a condition in which seeding activity can be maintained for a long period of time even when PrP-res is negative.

In contrast, the seeding activity of N2a58/22L was reduced to below the detection limit and did not recover after PPS removal. This result is consistent with previous reports indicating that PrP-res did not return after PPS treatment of scrapie-infected neuroblastoma cells^4^ or a transformed deer cell line persistently infected with chronic wasting disease followed by passaging in PPS-free medium^23^.

Although PPS inhibited the PrP^Sc^ conversion reaction in PMCA, this effect varied greatly depending on the strain, with much weaker inhibitory effects observed for FK1 and mBSE strains compared with scrapie-derived mouse adapted strains ME7, Chandler, and 22L. FK1 is a mouse-adapted strain of GSS (P102L mutation), a hereditary prion disease, while mBSE is a mouse-adapted strain of classical BSE. The fact that both these strains were resistant to PPS suggests a gap in the conversion mode between them and the three scrapie-derived strains. The results of PMCA may explain why low-level persistent prion infection keeping measurable seeding activity occurred only in N2a58/FK1. Specifically, heparin, a type of polyanion, binds to PrP^C^^28^ and promotes prion replication in PMCA^29^. PPS, a heparin analog, may compete with heparin or directly interact with PrP^C^ independently of heparin^30–33^ to inhibit prion replication in vitro and in vivo. Accordingly, differences in the final PrP^Sc^ structure of each prion strain led to differences in the conversion process of PrP^C^ bound to heparin and/or PPS, which may be the reason why the anti-prion effect of PPS was strain-dependent. Intraventricular infusion of PPS prolonged the survival of mice infected with RML (the alias of Chandler strain) or FK1, but prolongation of the FK1 incubation period was reportedly shorter than that of RML, demonstrating strain dependence of the anti-prion effect of PPS at the animal level^9^. Although the relevance to PPS is unclear, it has also been reported that, unlike Chandler and 22L, FK1-infected cultured cells exhibited significant changes in PrP-res in response to autophagy accelerators and inhibitors^34^, which may corroborate differences in conversion mode and PrP^Sc^ structure.

Clinical trials of PPS for patients with prion disease have been conducted in Japan^35^ and the UK^10,11^. Although there is a report of one case in which postmortem examination showed a significantly reduced level of PrP-res in the brain compared with levels in brains without PPS treatment^36^, there was no obvious improvement in clinical symptoms in any case of familial, ectopic, or sporadic CJD treated with PPS in Japan^35^. Notably, in two clinical studies of UK patients with variant CJD, three out of four^10^ and four out of five patients^11^ survived longer than those who did not receive PPS treatment. These differences may result from variations in the efficacy of PPS depending on the subtype of CJD. At present, it remains difficult to determine whether PPS is effective for treating patients with CJD because the number of patients is small and outcome criteria are unclear.

Previous screening has typically identified anti-prion compounds targeting inhibition of PrP-res accumulation in prion-infected cell cultures^37^. In this study, we demonstrated that seeding activity can remain even after PrP-res is lost in a strain-dependent manner. Therefore, the highly sensitive seeding activity measured by RT-QuIC is a useful indicator for screening of prion disease therapeutics in prion-infected cultured cells, whereas measurement of PrP-res levels alone is likely insufficient. Furthermore, the finding that different prion strains exhibit varying effects to drugs is yet another factor complicating the development of therapeutic agents for prion diseases^38–40^. Screening of prion-infected cells is limited by the difficulty of persistently infecting cells with certain prion strains, especially CJD. Our results show that even for prion strains that cannot be persistently infected in cultured cells, PMCA may be able to predict an inhibitory effect on the prion strain.

In conclusion, we have shown that PPS induces low-level persistent prion infection keeping measurable seeding activity without PrP-res detection in a strain-dependent manner.

## Methods

### Cell culture

The establishment of a mouse PrP-overexpressing neuroblastoma cell line (N2a58) and 22L-infected N2a58 cells (N2a58/22L) was described previously^41^. FK1-infected N2a58 cells (N2a58/FK1) were also described separately^42^. Cells were cultured at 37 °C in Dulbecco’s Modified Eagle’s Medium (Cat. No. 08458-16; Nacalai Tesque, Kyoto, Japan) containing 10% fetal bovine serum and penicillin–streptomycin (Cat. No. 26253-84, Nacalai Tesque) in a 5% CO_2_ incubator.

### Administration of Congo red, alprenolol, and PPS to prion-infected cells

Prion-infected N2a58 cells were plated in six-well plates and treated with 50 μM Congo red (Cat. No. 09403-02, Nacalai Tesque), 50 μM alprenolol (Skajilol, Kotobuki Pharmaceutical, Nagano, Japan) or 20 μg/ml PPS (Cartrophen Vet, Biopharm Australia, Bondi Junction, Australia). Concentrations of compounds added to cultured cells in this study referred to concentrations previously reported to reduce PrP-res in prion-infected cells^5,6,22^. Cells were passaged every 4 days and sampled each time for WB and RT-QuIC assays.

### Western blotting of cell lysates

Cell lysates were collected in lysis buffer [0.5% Triton X-100, 0.5% deoxycholic acid, 150 mM NaCl, and 50 mM Tris–HCl (pH 7.5)]. Total protein concentrations of cell lysates were quantified using a BCA Protein Assay Kit (#23225; Pierce, Waltham, MA, USA). Cell lysate samples were digested with 10 μg/ml PK at 37 °C for 30 min, subjected to sodium dodecyl sulfate polyacrylamide gel electrophoresis (SDS-PAGE) on NuPAGE 12% BisTris gels (Cat. No. NP0343; Life Technologies Corporation, Carlsbad, CA, USA), and transferred to polyvinylidene difluoride membranes (Cat. No. IPVH00010; Merck Millipore, Burlington, MA, USA). Membranes were subsequently treated with Blocking One (Cat. No. 03953-95, Nacalai Tesque) for 1 h at room temperature. T2 antibody, a horseradish peroxidase-conjugated anti-PrP monoclonal antibody that recognizes mouse PrP135-140^43^, was diluted 5000-fold and used as the primary antibody. Chemiluminescence was detected using EzWest Lumi plus (Cat. No. WSE-7120L; ATTO, Tokyo, Japan). Images were obtained by LuminoGraph I (WSE-6100, ATTO, Tokyo, Japan), a chemiluminescence imaging apparatus.

### RT-QuIC

Cells seeded for conversion reaction were dissociated from dish with 20 mM ethylenediaminetetraacetic acid (EDTA) and subjected to RT-QuIC. Numbers of cells were counted with a hemocytometer. Recombinant mouse PrP (rMoPrP 23–231) was purified as previously described^12^. Cells serially diluted with phosphate-buffered saline (PBS) were added to 95 μl of reaction buffer [500 mM NaCl, 25 mM PIPES (pH 7.0), 10 μM thioflavin T, 1 mM EDTA, and 0.001% SDS] in 96-well plates (Cat. No. 265301; Nunc, Rochester, NY, USA). The reaction was performed in samples alternately shaken and incubated for 30 s each using an Infinite F200 (Tecan, Männedorf, Switzerland). SD_50_ values were calculated using the Spearman–Kärber method.

### Quantification of the inhibitory effect of PPS on PMCA

Investigation of optimal conditions for PMCA using brain homogenates, especially sonication conditions, has been previously described^44^. ICR mouse brains were homogenized at 10% (w/v) in PMCA buffer (4 mM EDTA and 1% Triton-X100 in PBS) using a Beads Crusher (Beads Crusher μT-12; TAITEC, Saitama, Japan) and rotation at 4 °C for 1.5 h. PMCA samples were prepared by adding 5 μl of serial diluted PPS solution to 45 μl of 10% (w/v) ICR mouse brain homogenate with 0.01% (w/v) prion-infected mouse brain, 0.3 mg/ml heparin, and 0.05% digitonin. The mixture was loaded into 0.2-ml PCR tube eight-strips (Cat. No. SKPCR8D; Seiko, Tokyo, Japan) along with 2-mm zirconia beads (Cat. No. ZB-20; TOMY, Tokyo, Japan) and positioned on the floated plate holder of a Cup horn sonicator (Misonix-3000; Cole-Parmer, Vernon Hills, IL, USA). PMCA was performed for 16 h, consisting of 30 min incubation at 40 °C followed by a 20-s pulse of sonication. PMCA products were digested with 40 μg/ml of PK buffer and heated to 95 °C for 10 min in SDS sample buffer. SDS-PAGE was performed using 15% Tris–glycine gels. Western Blotting was then performed using the method described above. CS Analyzer (ATTO) was used to measure the intensity of the detected PrP-res bands; to quantify the inhibitory effect of PPS on PMCA and to compare between different blots, the intensity of PrP-res without PPS on the same membrane was set to 100%.

### Preparation of PrP^Sc^ seed for PMCA

Mouse-adapted scrapie strains 22L, Chandler, ME7, as well as mouse-adapted classical BSE (mBSE) were propagated in ICR mice. The mouse-adapted GSS strain Fukuoka-1 (FK1) was kindly provided by N. Nishida (Nagasaki University, Nagasaki, Japan). Preparation of prion-infected brain homogenates was previously described^29^.

### Ethics approval

ICR mice (11 weeks old, female) for PMCA experiments were obtained from SLC, Japan. No animal experiments were conducted in the experiments described in this manuscript. All experimental protocols using animal tissues were approved by the Animal Care and Use Committee of University of Miyazaki (approval ID: 2019-010) and were performed in accordance with relevant guidelines and regulations.

### Immunofluorescence assay

N2a58, N2a58/22L, and N2a58/FK1 cells were grown on four-well chambered slide cover glass (Cat. No. 154917, Nunc) and treated with 20 μg/ml PPS. Cells were washed with PBS and then fixed with pre-warmed 4% paraformaldehyde in PBS containing 4% sucrose for 10 min. After permeabilization with 0.1% Triton-X100 for 10 min, cells were treated with 5 M guanidine thiocyanate for 10 min and blocked with 5% fetal bovine serum in PBS. Next, cells were incubated with anti-PrP mouse monoclonal antibody 132 (mAb132) to detect PrP^45^. Alexa Fluor 488-conjugated monoclonal anti-mouse IgG (Cat. No. A11017, Life Technologies Corporation) was used as the secondary antibody. Cell nuclei were counterstained with 4′,6-diamidino-2-phenylindole.

### Statistical analysis

For WB after PMCA, statistical significance was calculated by Dunnett's multiple comparison test using GraphPad Prism (GraphPad Software, San Diego, CA, USA).

## Supplementary Information

Supplementary Figures.

## Data Availability

The datasets used and/or analyzed during the current study available from the corresponding author on reasonable request.
