# Supplementary material for: Pentosan polysulfate induces low-level persistent prion infection keeping measurable seeding activity without PrP-res detection in Fukuoka-1 infected cell cultures
Source: Sci Rep. 2022 May 13;12:7923. doi: 10.1038/s41598-022-12049-z (PMC9106670; doi:10.1038/s41598-022-12049-z)
Supplement: Supplementary file 1 — Supplementary Figures. [file 41598_2022_12049_MOESM1_ESM.pdf]

Pentosan polysulfate induces low-level persistent prion infection keeping measurable seeding activity without PrP-res detection in Fukuoka-1 infected cell cultures

Hanae Takatsuki, Morikazu Imamura, Tsuyoshi Mori, Ryuichiro Atarashi

Supplementary Information

Supplementary Figure 1

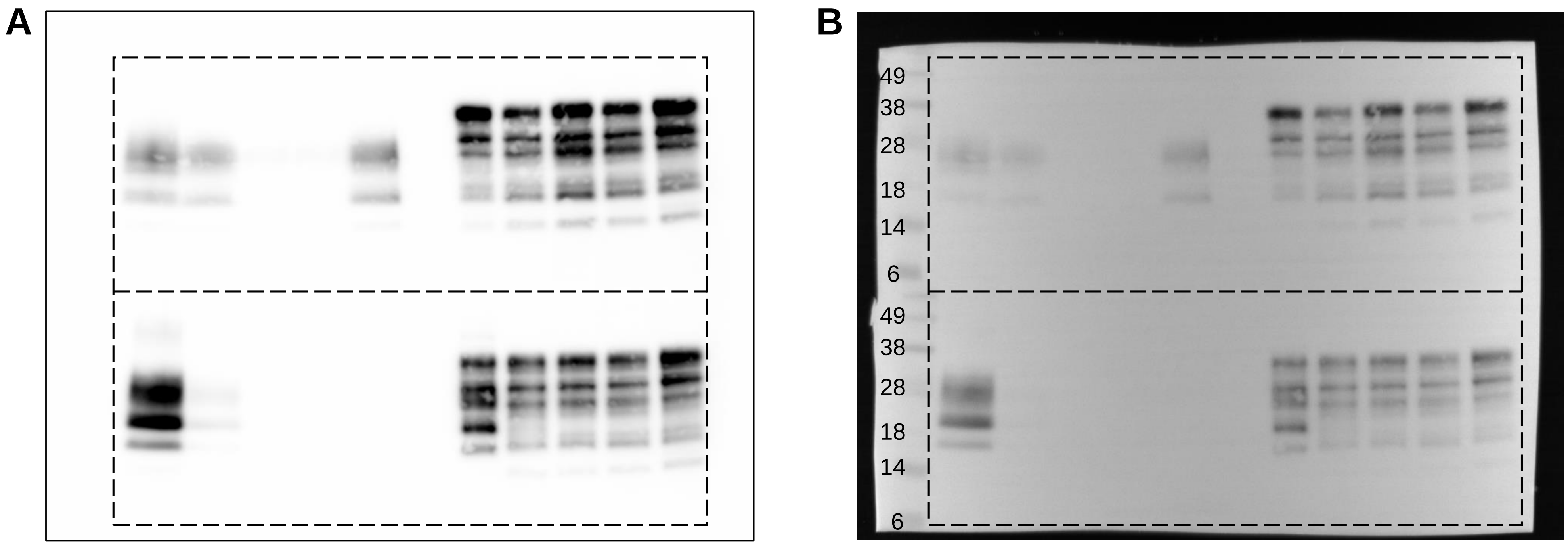

(A) The full length blot of the cropped image shown in Figure 4A. Two gels (placed at the top and bottom) were transferred to a single polyvinylidene difluoride membrane for Western blotting. **(B)** Merged images of marker imaging and luminescence images. The numbers represent the molecular weight of the marker.

Supplementary Figure 2

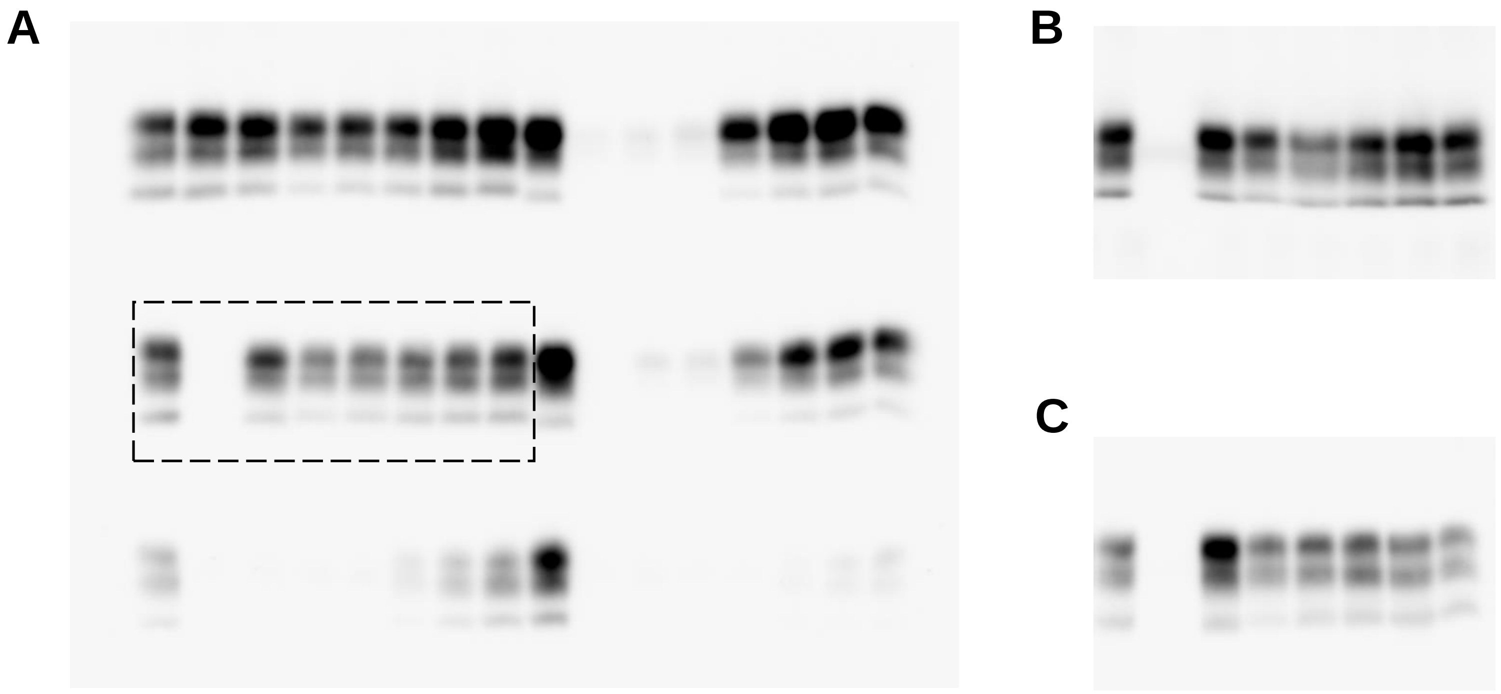

Inhibitory effect of PPS on PMCA seeded with FK1. (A) The full length blot of the cropped image shown in Figure 5A. Three gels (placed at the top, middle, and bottom) were transferred to a single polyvinylidene difluoride membrane for Western blotting. **(B)** Blot image of Experiment 2. **(C)** Blot image of Experiment 3.

Supplementary Figure 3

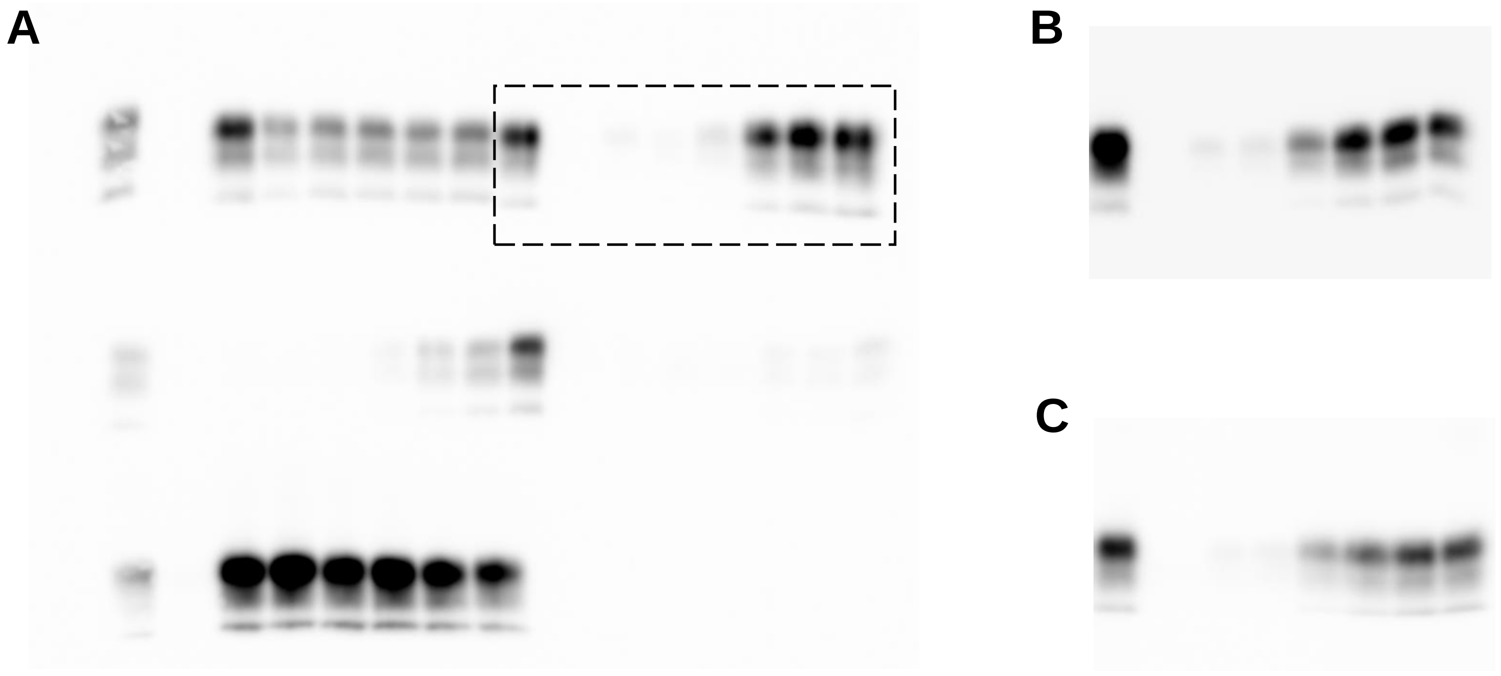

Inhibitory effect of PPS on PMCA seeded with 22L. (A) The full length blot of the cropped image shown in Figure 5A. Three gels (placed at the top, middle, and bottom) were transferred to a single polyvinylidene difluoride membrane for Western blotting. **(B)** Blot image of Experiment 2. **(C)** Blot image of Experiment 3.

Supplementary Figure 4

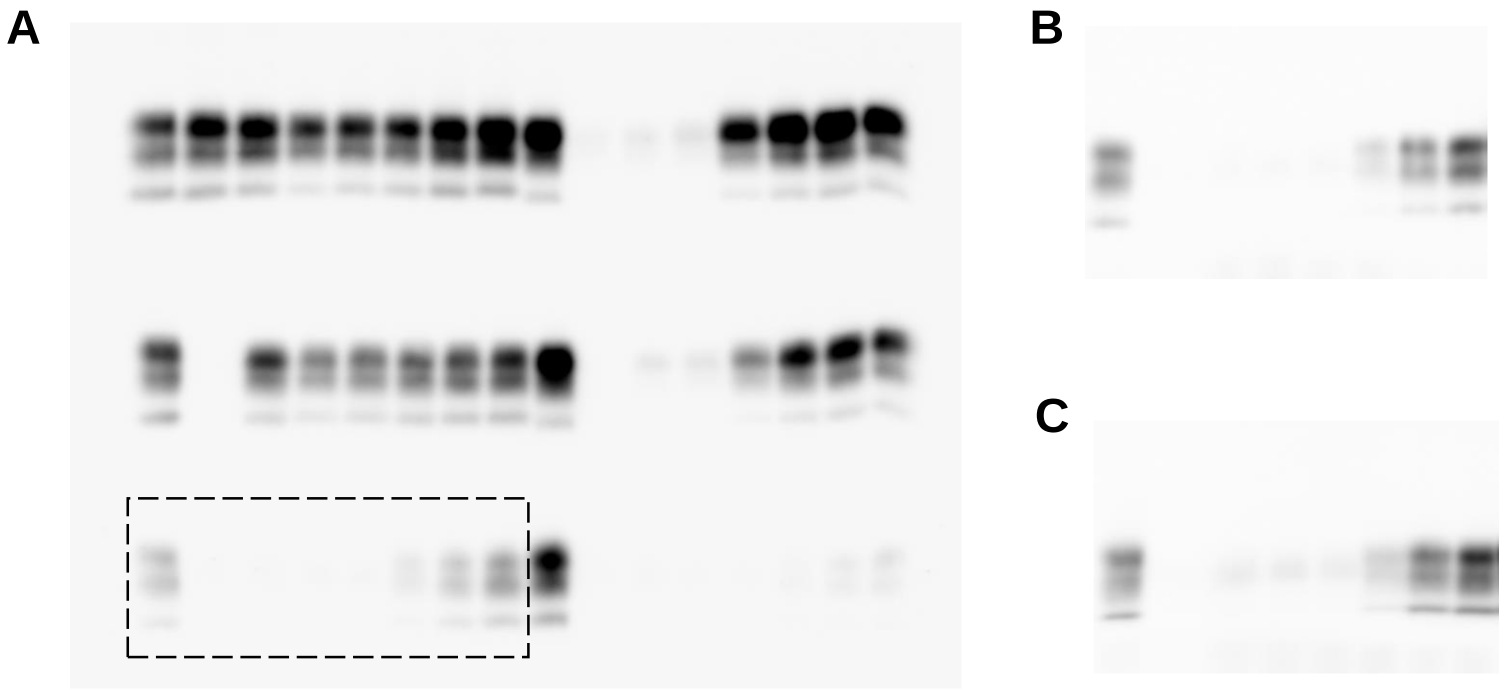

Inhibitory effect of PPS on PMCA seeded with Chandler. (A) The full length blot of the cropped image shown in Figure 5A. Three gels (placed at the top, middle, and bottom) were transferred to a single polyvinylidene difluoride membrane for Western blotting. **(B)** Blot image of Experiment 2. **(C)** Blot image of Experiment 3.

Supplementary Figure 5

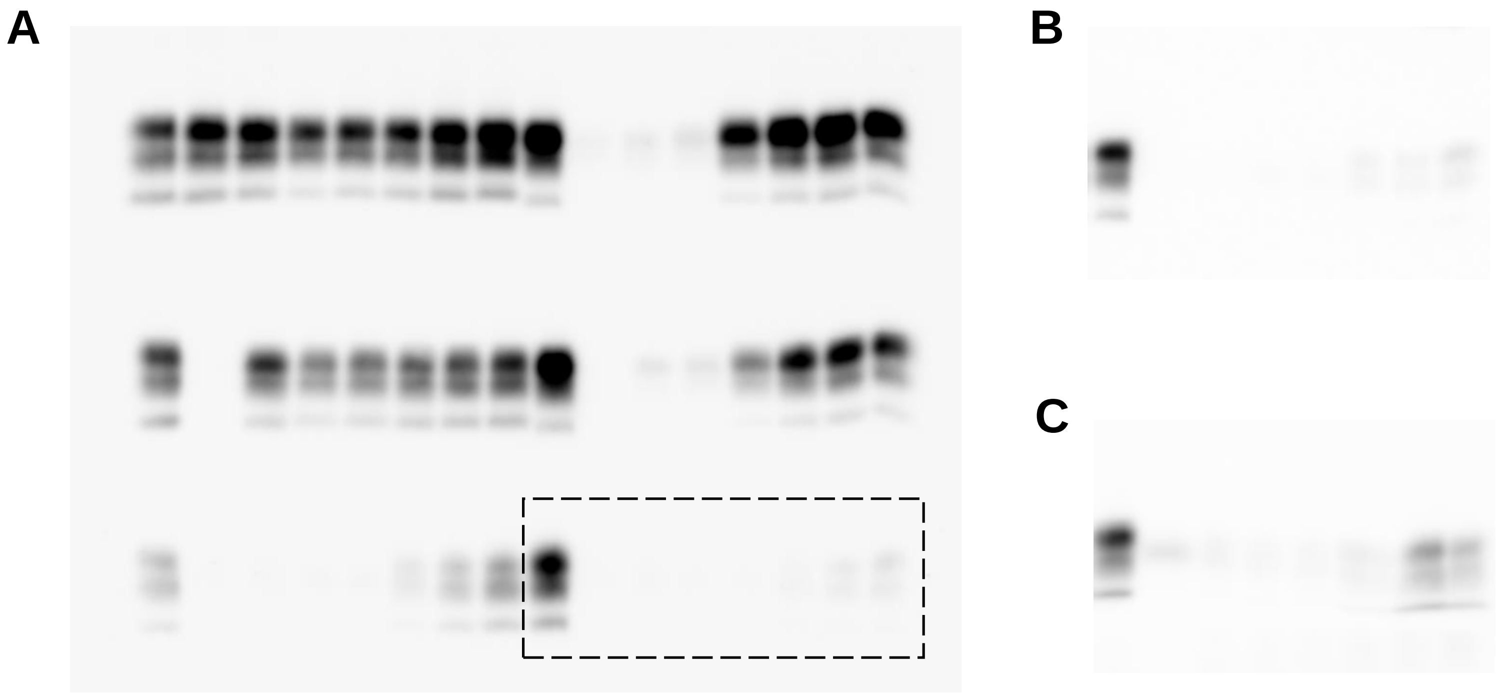

Inhibitory effect of PPS on PMCA seeded with ME7. (A) The full length blot of the cropped image shown in Figure 5A. Three gels (placed at the top, middle, and bottom) gels were transferred to a single polyvinylidene difluoride membrane for Western blotting. **(B)** Blot image of Experiment 2. **(C)** Blot image of Experiment 3.

Supplementary Figure 6

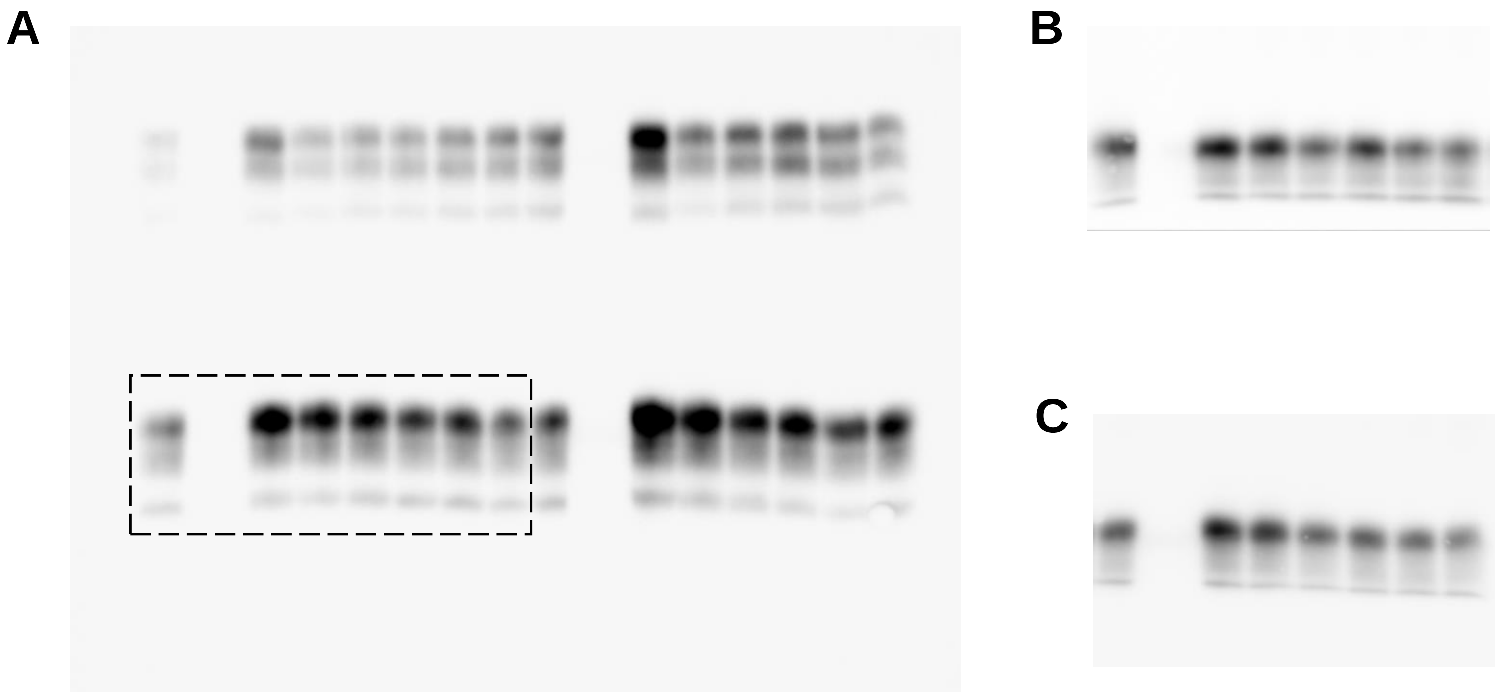

Inhibitory effect of PPS on PMCA seeded with mBSE. (A) The full length blot of the cropped image shown in Figure 5A. Two gels (placed at the top and bottom) were transferred to a single polyvinylidene difluoride membrane for Western blotting. **(B)** Blot image of Experiment 2. **(C)** Blot image of Experiment 3.
